# Delivering colon cancer survivorship care in primary care; a qualitative study on the experiences of general practitioners

**DOI:** 10.1186/s12875-021-01610-w

**Published:** 2022-01-17

**Authors:** Julien A. M. Vos, Robin de Best, Laura A. M. Duineveld, Henk C. P. M. van Weert, Kristel M. van Asselt

**Affiliations:** 1 Department of General Practice, Amsterdam UMC, University of Amsterdam, Postbox 22660, Amsterdam, 1100 DD the Netherlands; 2 Program of Personalized Medicine & Quality of Care, Amsterdam Public Health Research Institute, Amsterdam, the Netherlands

**Keywords:** Qualitative research, Implementation sciences, Cancer survivorship care, Colon Cancer, Primary health care, General practitioners, Normalisation process theory

## Abstract

**Background:**

With more patients in need of oncological care, there is a growing interest to transfer survivorship care from specialist to general practitioner (GP). The ongoing I CARE study was initiated in 2015 in the Netherlands to compare (usual) surgeon- to GP-led survivorship care, with or without access to a supporting eHealth application (Oncokompas).

**Methods:**

Semi-structured interviews were held at two separate points in time (i.e. after 1- and 5-years of care) to explore GPs’ experiences with delivering this survivorship care intervention, and study its implementation into daily practice. Purposive sampling was used to recruit 17 GPs. Normalisation Process Theory (NPT) was used as a conceptual framework.

**Results:**

Overall, delivering survivorship care was not deemed difficult and dealing with cancer repercussions was already considered part of a GPs’ work. Though GPs readily identified advantages for patients, caregivers and society, differences were seen in GPs’ commitment to the intervention and whether it felt right for them to be involved. Patients’ initiative with respect to planning, absence of symptoms and regular check-ups due to other chronic care were considered to facilitate the delivery of care. Prominent barriers included GPs’ lack of experience and routine, but also lack of clarity regarding roles and responsibilities for organising care. Need for a monitoring system was often mentioned to reduce the risk of non-compliance. GPs were reticent about a possible future transfer of survivorship care towards primary care due to increases in workload and financial constraints. GPs were not aware of their patients’ use of eHealth.

**Conclusions:**

GPs’ opinions and beliefs about a possible future role in colon cancer survivorship care vary. Though GPs recognize potential benefit, there is no consensus about transferring survivorship care to primary care on a permanent basis. Barriers and facilitators to implementation highlight the importance of both personal and system level factors. Conditions are put forth relating to time, reorganisation of infrastructure, extra personnel and financial compensation.

**Trial registration:**

Netherlands Trial Register; NTR4860. Registered on the 2nd of October 2014.

**Supplementary Information:**

The online version contains supplementary material available at 10.1186/s12875-021-01610-w.

## Background

Colon cancer is among the five most prevalent types of cancer [1]. Due to improvements in the detection, diagnosis and treatment of colon cancer, an increasing number of cancer survivors are entering a survivorship care phase in which the focus shifts from the initial specialist treatment to rehabilitation, management of other sequelae and recurrence detection [2].

Different survivorship care models exist [3], but there is little data to support any given type. In the Netherlands all colon cancer patients remain in secondary care to receive survivorship care by a surgeon or specialised oncology nurse in the hospital. The traditional strengths of primary care - including its continuous, coordinated and comprehensive care for individuals - may lend themselves for the improvement of survivorship care, and therefore general practitioners (GPs) might play a more prominent role [4, 5]. Survivorship care in primary care has shown similar effects on clinical and patient-reported outcomes, while resulting in lower costs [6, 7]. Though a greater role by the GP is supported by many patients and healthcare professionals, there have also been some reservations [8–10]. These uncertainties formed the basis for the I CARE study (Improving Care After colon canceR treatment in the Netherlands, personalised care to Enhance quality of life), comparing GP- to surgeon-led survivorship care for colon cancer patients, with or without access to a supporting e-Health application (Oncokompas) [11]. Within the first year after surgery, the I CARE study found no important differences in QoL changes between trial arms, demonstrating the potential of GP-led survivorship care as an alternative to hospital-based care [12].

Previous research has recognised the importance of carrying out a process evaluation for complex healthcare interventions such as these [13, 14]. The Normalisation Measure Development (NoMAD) questionnaire is one of the tools that can be used to measure implementation processes from the perspectives of healthcare professionals [15, 16], but it needs to be adapted to fit the purpose of the intervention and target the responders of the survey. Qualitative data helps to capture emerging changes in the implementation of an intervention, and may be used at different points in time [13]. This qualitative study formed the first step in the mixed-methods process evaluation of the I CARE study.

## Methods

### Aim

Parallel to the trial, semi-structured interviews were held to explore GPs’ experiences with the delivery of a colon cancer survivorship care intervention, and study its implementation into clinical practice. After 1-year of care, exploratory interviews were held in order to identify, anticipate and manage possible barriers or shortcomings early on in the intervention. After 5-years of care, theory-driven interviews were held to help understand how the intervention was implemented, embedded and sustained in primary care. Because the aims after 1- and 5-years of care differed, this was not intended as a qualitative longitudinal study.

### Intervention design and setting

Eight Dutch hospitals participated in the multi-centre 2 × 2 factorial randomised controlled I CARE study [11]. The primary outcome is quality of life. Secondary outcomes include (among others) recurrence detection, number of referrals and economic evaluation. Patients who were surgically treated with curative intent for stage I-III colon cancer were considered eligible and randomly assigned into four groups comparing survivorship care by a surgeon to care by a general practitioner (GP), with or without access to a supporting eHealth application (Oncokompas). This eHealth application aims to support cancer patients in finding and obtaining supportive care by providing personalized feedback [17]. Patients were granted access to the application, but use was not otherwise endorsed. Eligible patients were recruited by their treating physicians, after which their GPs were asked for consent. The national colon cancer follow-up guideline was summarized in a survival care plan (SCP) and provided to the GPs who were randomised to deliver survivorship care. The SCP was not part of the intervention, and therefore did not contain any personalized information or recommendations for the patients, but included general information on the follow-up schedule, disease symptoms, treatment side effects, use of the distress thermometer as a tool to screen for psychosocial distress and recommendations for healthy lifestyle [18, 19]. Patients were referred to their GP for survivorship care after curative treatment in the hospital was finished (shortly after surgery or after adjuvant chemotherapy). Inclusion of patients lasted from March 2015 to November 2018. In total, 303 patients were included. The date of the 5-year follow-up completion is by the end of 2023.

### Sampling strategy

GPs who delivered survivorship care were invited by email and then telephone to elaborate on their experiences. A purposive sampling strategy was employed. The invitation was based on patients’ tumour stages (due to the varying intensities and complexities of follow-up schedules). Participation was on a voluntary basis. Out of 27 GPs invited for an interview, 17 consented to participate. Reasons to decline participation related to having no interest or time. There were no prior relationships of interviewers with participants. Because only few GPs had completed the 5-year follow-up programme at the time of this study, three GPs participated in both interviews after 1- and 5-years of care.

### Data collection and processing

After 1-year of care, exploratory interviews were held by a research assistant (Ms. AS-J, research assistant). Though the exploratory interviews were not based on an existing theory, framework, or model of implementation sciences, they included many questions regarding the execution of the intervention (Additional file 1: appendix S1). The exploratory interviews therefore helped to understand how the intervention was delivered, and provided additional data for this process evaluation.

After 5-years of care, theory-driven interviews were conducted by one or two independent researchers (Mrs. RdB, medical student at the time of the study, and Mr. JV, PhD candidate), who had training in qualitative research. A semi-structured interview guide was developed based on Normalisation Process Theory (NPT). NPT provides a flexible and pragmatic framework to help understand how interventions are implemented, embedded and sustained in complex healthcare settings, including primary care [20–22]. NPT is comprised of four core constructs (i.e. coherence, cognitive participation, collective action and reflexive monitoring) and sixteen components which can be modified and adapted to fit the purpose of the intervention. A description of NPT core constructs and components relevant to the I CARE study can be found in Table 1. When patients had randomised for access to Oncokompas, additional questions were asked about the GPs’ awareness of this application and its influence on the care process. The interview guide was pilot tested with 2 GPs, after which minor adjustments were made to optimise and finalise the interview guide (Additional file 1: appendix S2).

**Table 1:** Description of the core constructs and components of Normalisation Process Theory (NPT)

Construct	Description
Coherence	*Sense-making work*
- Differentiation	How does the intervention differ from the current practice?
- Individual specification	Do GPs understand what tasks are required of them to deliver the intervention?
- Communal specification	Do GPs have an understanding about the purpose of the intervention?
- Internalisation	What added value or benefits can be derived from the intervention?
Cognitive participation	*Relational work*
- Initiation	What motivated GPs to participate in the intervention?
- Enrolment	Do GPs believe they are the correct professional to drive forward the intervention?
- Legitimation	Do GPs believe it is appropriate for them to be involved in the intervention?
- Activation	What could GPs do together with other stakeholders to sustain the intervention?
Collective action	*Operational work*
- Interactional workability	What is the interactional work that GPs do to deliver the intervention?
- Skill set workability	Do GPs have the correct skills and training to deliver the intervention?
- Relational integration	Do GPs have confidence in delivering the intervention?
- Contextual integration	How is the intervention incorporated into local and national resources and policies?
Reflexive monitoring	*Appraisal work*
- Systematisation	What are the GPs judgements regarding the usefulness of the intervention?
- Individual appraisal	What is the GPs individual appraisal regarding the intervention?
- Communal appraisal	How do GPs collectively judge the effectiveness of the intervention?
- Reconfiguration	What are the GPs recommendations to modify and enhance the intervention?

All interviews were held on-site (exploratory interviews after 1-year), or through videoconferencing and telephonically due to the security measures surrounding the COVID-19 pandemic (theory-driven interviews after 5-years). No field notes were made. The interviews were audio-taped and transcribed verbatim. Transcripts were verified against the original audio data (Mrs. RdB). After transcription, all audio data were erased, and transcripts were anonymised. The interviews lasted between 18 and 51 min. The consolidated criteria for reporting qualitative studies (COREQ) checklist was used for the reporting of this study (Additional file 1: appendix S3) [23].

### Data analysis

Because the aims and interview guides after 1- and 5-years differed, thematic analysis was chosen. Coding and identification of themes was performed by two independent researchers (Mrs. RdB and Mr. JV) using an iterative approach [24]. Discrepancies between codes and themes were discussed between the two researchers. In case of disagreement, a third party (Ms. KvA, PhD) was consulted to reach consensus. Data saturation was assumed when no new themes were identified after eight consecutive transcripts [25]. Thematic analysis resulted in overlapping themes after 1- and 5-years of care, after which the data from the interviews was pooled. NPT was subsequently used as a way to describe and order the data. Regular debriefing sessions were held with other research members to reflect on study processes and discuss results. Detailed records were held of these discussions and decisions. Transcripts were coded using MAXQDA Plus 2020 Network Software [26].

### Rigour of the study

The use of two different interview aims and guides may lead to questions regarding the validity of the results. Strategies to improve validity included; investigator triangulation, purposive sampling (taking into account the varying intensities and frequencies of follow-up), pilot testing of the theory-driven interview guide, providing qualitative training for interviewers and establishing an audit trail. Reliability was improved through recursive and repetitive checks, and regular debriefing sessions. The combined use of both exploratory and theory-driven interviews will have helped to portray the experiences that are lived and perceived over the course of the intervention.

## Results

### Participants

In total, 20 interviews were held with 17 GPs. Table 2 shows the characteristics of the participating GPs. The participating GPs delivered care for 16 patients who had a mean age of 65.7 years (SD 7.8), male sex (*n* = 9), stage II-III colon carcinoma (*n* = 10), received adjuvant chemotherapy (*n* = 3) and had access to Oncokompas (*n* = 9). One GP had taken over survivorship care from another GP after the first year. During follow-up, one patient was diagnosed with primary rectal cancer with lymph node metastases after 2 years of care for which he was referred back to the surgeon.

**Table 2:** General practitioner characteristics

	*Exploratory interviews after 1-year of care (n = 7)*^a^	*Theory driven interviews after 5-years of care (n = 10)*
Age in years (mean, SD)	46.1 (10.4)	46.4 (8.5)
Sex, male (n, %)	3 (43%)	4 (40%)
Years of experience as a GP (median, range)	14 (16)	13 (11)
Years working in same practice (median, range)	10 (18)	10 (14)
Self-employed (n, %)	5 (71%)	7 (70%)

^a^Questions about the characteristics were not part of the exploratory interviews. Efforts were made to gather these details through email and telephone. Three GPs could not be reached.

### Coherence: making sense of survivorship care

Coherence referred to the question whether delivering colon cancer survivorship care differed from current clinical practice, and whether GPs had an understanding of what was required of them, the purpose and potential benefits. Overall, GPs expressed that cancer survivorship care is not complex and dealing with cancer repercussions is already part of their current work. GPs used the survival care plan (SCP) at the beginning of the intervention, but found it to have little added value later on. Nevertheless, there was a common request among GPs for additional education regarding post treatment symptoms, side-effects and how to handle them after both 1- and 5-years of care.

GPs often felt responsible for the execution of survivorship care (i.e. ordering follow-up tests), while they felt that the patient was primarily in charge of making follow-up appointments. At other times it felt as a form of shared care.

*“I think this was a very good example of shared care. We went through that schedule together, and we looked at what had to be done”* (female, 5 years of care).

GPs readily identified advantages for patients, such as the proximity to home, familiar surroundings, limited waiting time, less negative connotation than the hospital and increased attention to other aspects of care. From a social perspective, the decreases in healthcare costs were mentioned. Advantages for the GP included their increased contact with the patient and having a better grasp on the patients’ condition.

*“I felt more involved [with the patient], I do believe that through delivering cancer survivorship care there can be a deepening of the relationship”* (male, 5 years of care).

On the other hand, GPs reported concerns about taking away the patients’ right to choose secondary care, timely referrals and less prompt care in primary care (with regards to additional testing). The disadvantages for the GPs centred around their lack of experience and routine with this type of care. Table 3 shows an overview of the (perceived) barriers and facilitators mapped out to NPT constructs. Most barriers and facilitators remained stable over time as they were mentioned after both 1- and 5-years of care.

**Table 3:**
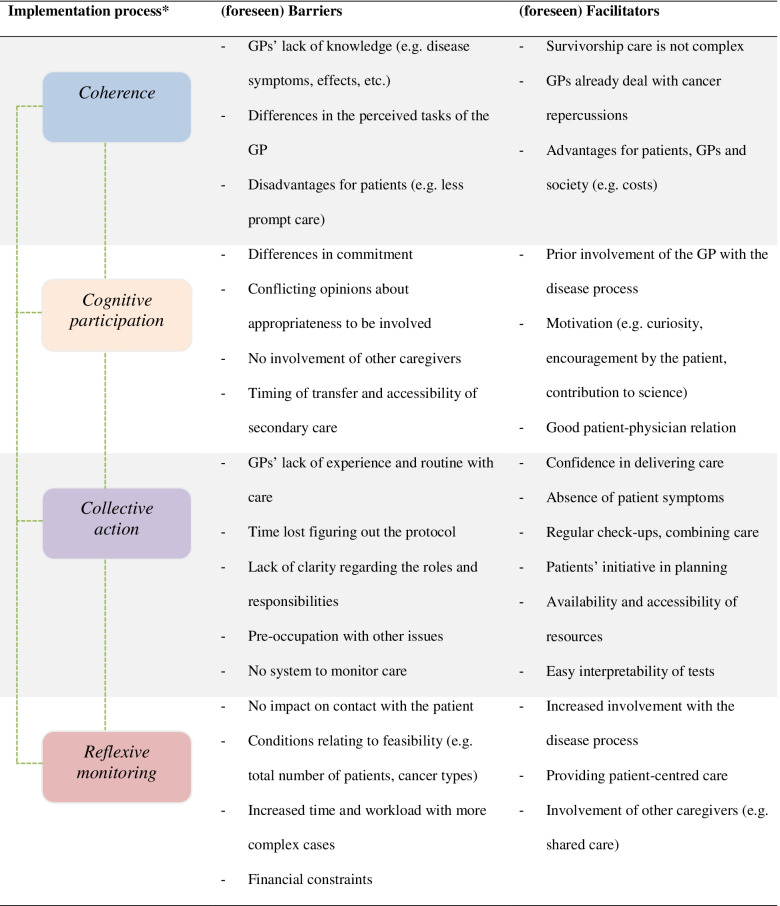
Overview of barriers and facilitators mapped out to Normalisation Process Theory (NPT) constructs

* For NPT it is often assumed that its mechanisms operate simultaneously, rather than sequentially [1]. Therefore, barriers and facilitators can interact dynamically and play a role in the different stages of the implementation process.

References

1. May CR, Cummings A, Girling M, et al. Using Normalization Process Theory in feasibility studies and process evaluations of complex healthcare interventions: a systematic review. Implement Sci. 2018;13(1):80.

### Cognitive participation: investment in survivorship care

Cognitive participation addressed the question whether GPs were motivated to deliver colon cancer survivorship care and believed it was appropriate for them to be involved. Most GPs were involved with the disease process prior to the intervention. Knowledge of the patients’ context, and also good patient-physician relationship were mentioned to facilitate the delivery of care (Table 3). Both intrinsic and extrinsic reasons to participate were mentioned. Intrinsic reasons related to the curiosity of the GP, challenging aspect of the intervention and personal experience with or preference for care. The encouragement by their patients’ preference for GP-led care and importance of medical research were mentioned as extrinsic reasons.

Opinions and beliefs about delivering survivorship care in primary care were conflicting. There were GPs who committed to the intervention and would recommend it to other colleagues.

*“It’s nice that this type of care can also be delivered in primary care, and I think it’s also a stepping stone since [ … ] cancer is becoming more of a chronic disease in secondary care”* (male, 5 years of care).

These GPs also believed it was appropriate for them to be involved in survivorship care. Others did not think the intervention constituted a constructive part of a GPs job and would not recommend it to colleagues.

*“I don’t consider this a physicians’ task [ … ] I’m a physician, so I don’t believe I should spend my time on cookbook medicine”* (female, 5 years of care).

In order to adopt and sustain the intervention into clinical practice, it was deemed necessary that treatment in the hospital was finished, patients did not experience any complications, and access to secondary care was guaranteed at all time. The majority of GPs did not involve other caregivers from their practice. Two GPs involved a practice assistant in the requesting of follow-up tests and appointments. GPs were willing to involve and engage others, but this was not considered worthwhile for just one patient.

“*If it’s only one patient, you don’t involve a physical therapist, a psychologist, an occupational therapist, or whatsoever [ … ] but if it’s a larger group of patients on a structural basis, than it obviously makes sense”* (male, 1 year of care).

### Collective action: delivering survivorship care in practice

Delivering colon cancer survivorship care required GPs to do new things and interact differently with their patients. Collective action referred to the question whether GPs had the correct skills, training and confidence to do so. During consultations, GPs discussed physical and psychosocial wellbeing. Patient wellbeing was the main point of focus during the earlier stages of the intervention, while later on the consultations centred around test results. Patients often had little physical or psychosocial needs, and therefore a functional approach to survivorship care seemed sensible, even within the first year after surgery.

*“I ask her; how are you doing? I tell her; the CEA test result is good [ … ] So actually the follow-up consultation constitutes very little. Look, if there was anything else, anxiety after cancer, or problems or questions regarding bowel movements or whatsoever, then it would constitute more of course, but she doesn’t have any problems”* (male, 1 year of care).

Lifestyle and preventive measures were often discussed in a broader context than the one specifically for colon cancer. None of the GPs discussed the use of Oncokompas or other supporting eHealth applications. Earlier consultations often took more time, but as time went on, GPs became more familiar with the intervention. Within the first year of the intervention, some of the GPs booked double consultation time to deliver care, while later on a single consultation was generally considered sufficient.

*“Everything was new and we sat together to check the follow-up schedule. It just took us some time to get used to it, how we were going to organise it, and what we had to talk about”* (female, 1 year of care).

A few GPs chose to schedule care ahead 1-year at the time, while others waited for the patient to contact the general practice. Patients’ initiative with respect to planning was considered to facilitate care. A lack of clarity regarding the responsibilities for organising follow-up tests by patient and GP was often perceived as a barrier and possible risk of non-compliance (Table 3).

*“The question remains; who is responsible? Does it remain with the patient to go for a follow-up test or does the physician have to keep reminding the patient?”* (male, 5 years of care).

These perceived responsibilities might as well change over time.

*“During the first year it was always the patient who would mention a month in advance ‘it’s time for my ultrasound’, while lately I have been the one saying ‘perhaps you should do another blood test again’. So I’ve noticed that the patient is less on top of it”* (female, 5 years of care).

Delivering colon cancer survivorship care in primary care was not deemed difficult. However, GPs were not able to remember the follow-up schedule and had to look up the schedule during each consultation. Time was lost figuring out the protocol and contacting secondary care physicians. This was mentioned after both 1- and 5-years of care. Concerns with other issues, such as patients’ comorbidities, was perceived as a barrier. The absence of patient symptoms and patients who already came for regular check-ups were considered to make care easier. Some GPs therefore chose to combine survivorship care with other types of chronic care.

Most GPs felt confident in delivering survivorship care, though some doubted their experience with this type of care, knowledge and capabilities.

*“In a general practice there are not that many patients with colon cancer who are in this phase. Even if you were to include all the patients from my practice, then it still wouldn’t be sufficient to gain any real routine”* (male, 5 years of care).

GPs sometimes mentioned the study participation in their agenda or the patient file as a reminder, but the GPs had no other system in place to monitor care. One GP mentioned that the research team served as a reminder, as they called every now and then to enquire about test results. To reduce the risk of non-compliance, a monitoring system like the one in place for chronic disease management was suggested as a possible solution.

Easy access to secondary care, close proximity to lab and imaging, and unambiguous test results facilitated survivorship care. On the other hand, a lack of transfer of information from secondary to primary care hindered the delivery of care.

### Reflexive monitoring: appraising the impact of survivorship care

With respect to reflexive monitoring, GPs were asked to appraise the intervention, reflect on its usefulness and possible embedding in general practice care. GPs generally appreciated delivering survivorship care. There were GPs who felt more involved with the disease process and had more frequent contacts with the patient, while others believed the intervention did not have any impact on their relationship with the patient (Table 3). GPs recognised the value of the intervention and considered it a good initiative with respect to the research objective (improving patient-centred care).

*“It is an opportunity to talk about how things are going, that is the nice thing this system. You can provide real personal continuity as much as possible”* (female, 1 year of care).

There was no consensus about transferring survivorship care from secondary to primary care on a permanent basis. Some GPs believed it would be feasible to deliver survivorship care, but conditions were put forth, such as a maximum number of patients and limited selection of cancer types. Other GPs did not believe it would be feasible due to the increases in workload, time and financial constraints, regardless of the timing of the interview.

*“When it comes to substitution of any kind, it is always said that ‘the GPs can do it’ [ … ] but there is no further discussion on how we are supposed to take over with the staff and time that we have, let alone that there ought to be financial compensation”* (female, 5 years of care).

Interestingly, one of the GPs that participated in both interviews, previously stated that it would not be feasible to deliver survivorship care for all colon cancer patients, but changed this opinion after 5-years by mentioning that survivorship care was not more time consuming than the care for a patient with diabetes. Recommendations were made in order to sustain colon cancer survivorship care in primary care. GPs would benefit from additional time and financial compensation, since more complex cases will also take additional time. The involvement of other caregivers, such as the practice nurse, physical therapist and psychologist, was suggested to improve survivorship care. Alternative strategies to care by a GP were also proposed, including shared forms of care with the specialist and care by a specialised oncology nurse.

*"A specialised nurse in the hospital can do it just as well as I can, plus they're cheaper and have closer bonds with the surgeons"* (female, 5 years of care).

## Discussion

### Summary

This semi-structured interview study explored the experiences of GPs with the delivery of a survivorship care intervention for colon cancer patients. Though many GPs recognised the potential benefit of the intervention (coherence), differences were seen in whether they considered survivorship care as belonging to their professional tasks (cognitive participation). Overall, delivering colon cancer survivorship care was not deemed difficult, but it required GPs to put in additional time and work (collective action). There was no consensus among GPs about a possible transfer of colon cancer survivorship care from secondary to primary care (reflexive monitoring). Conditions were put forth relating to time, finances and reorganisation of care. Using NPT as a conceptual framework, this study provided a better understanding of the barriers and facilitators to adopt the intervention into clinical practice. Barriers and facilitators reflected the full range of NPT constructs and highlighted the importance of both personal and system level factors.

### Comparison with existing literature

A previous review on the perspectives of GPs in the provision of follow-up cancer care, has demonstrated similar concerns raised in this study, such as the time investment, increased workload and the lack of experience and confidence of GPs [9]. Though Meiklejohn et al. concluded that GPs (and patients) supported a greater role by the GP, ‘there was some variation across studies with regard to interest in providing and beliefs about who GPs felt was best placed to provide follow-up cancer care’. However, most of these studies were based on GPs’ perspectives and preferences for cancer survivorship care, therefore illustrating expectations rather than experiences. Two previous trials on GP-led colon cancer survivorship care did not address the experiences of participating GPs [27, 28]. This semi-structured interview study provided new evidence on whether the intervention could become part of routine general practice care. Different strategies were chosen to organise survivorship care, in which sometimes the GP was in the lead, while at other times it was the patient or a shared form of care. A lack of clarity regarding the organisation of care was perceived as a barrier and possible risk of non-compliance, illustrating the importance of defining roles and responsibilities. In comparison to hospital-based survivorship care, in which follow-up consultations are usually limited to 10 min, GPs often had more time, especially within the first year of care. Nevertheless, GPs would still benefit from additional time. A possible explanation could be the comprehensiveness of care delivered by the GP [4, 5]. This may also be reflected by the content of survivorship care, in which GPs discussed not only physical and psychosocial wellbeing, but also lifestyle, preventive measures, and other chronic disease management.

GPs have showed positive attitudes towards providing eHealth applications, such as Oncokompas, to their patients [10]. Some GPs have even expressed the wish to be more involved with their patients' use of eHealth. Nevertheless, there was no awareness among GPs in this study of their patients’ use of Oncokompas. This could be explained by a majority of GPs following the needs of their patients to discuss the use of eHealth [10].

### Strengths and limitations

Purposive sampling created a representative group of GPs who delivered varying intensities and complexities of follow-up schedules for colon cancer patients. The large amount of data provided at two separate moments in time contributed to the solidity of the results. The use of an iterative analysis approach based on NPT further increased the robustness of the findings.

Challenges were faced in the recruitment of the I CARE study, resulting in an extended recruitment period [29]. Inherent to the I CARE study, selection bias of both patients and GPs could not be excluded.

*“The patient did choose to do it this way, so that’s an obvious bias”* (female, 5 years of care).

Reasons for patients and GPs to decline participation often related to the research objective and preferences for care, which may have led to an overrepresentation of participating patients and GPs who were generally positive about the intervention. Despite this observation, this study showed conflicting opinions and beliefs among GPs. It is possible that GPs with a more neutral opinion were less likely to agree to an interview than those with a strong opinion (positive or negative). These strong opinions can be considered a strength as they were more likely to reveal the experiences from either side of the spectrum. Though this was not intended as a longitudinal study, 3 GPs participated in both interviews. Interestingly, one of these GPs had changed his opinion about the intervention over time, highlighting the value of longitudinal qualitative research to help understand how and why experiences change.

### Implications for research and/or practice

This qualitative study provided the necessary data to customise the NoMAD for the I CARE study [15, 16]. The customised NoMAD will be distributed among all participating GPs at the end of the I CARE study to further assess the impact of the intervention on primary care and judge its implementation potential. Other stakeholders, including patients, specialists and healthcare policy makers, are evenly important in the decision on future practices, so further research should also focus on their experiences, opinions and beliefs. The optimal choice of survivorship care model is also likely to depend on the context and setting [3]. In the Netherlands, all residents have assured access to secondary care through universal healthcare coverage. GPs refer to secondary care following guidelines and agreements with healthcare insurance. The implications of these findings may therefore differ for countries with other healthcare systems. Different survivorship care models, including care by a specialised oncology nurse and shared forms of care, may provide a different opportunity and anticipate shortages in oncology workforce [6].

In this qualitative study, GPs’ opinions and beliefs about a possible future role in colon cancer survivorship care were conflicting. Based on the GPs’ experiences with this type of care, recommendations for future practices were established. To help improve GPs’ confidence and expertise on the subject, additional education is requested regarding treatment side-effects and symptoms. Changes need to be made to the infrastructure of the general practice so that it may be better equipped to accommodate care. These changes include the need for a monitoring system and the involvement and training of other caregivers, such as the physical therapist and psychologist. The role of paramedics working in primary care is already expanding, and joint collaborations could alleviate some of the work for the GP. To safeguard the continuity of care, clear agreements need to be in place with secondary care in case of a suspected recurrence, so that timely referral remains possible. And finally, in order for this alternative to hospital-based care to be sustainable, additional time and financial compensation is deemed necessary.

## Conclusions

GPs’ opinions and beliefs about a possible future role in colon cancer survivorship care vary. In order to adopt and sustain the intervention into routine practice, conditions and recommendations are put forth. Further research is needed to evaluate its implementation potential, including the perspectives of patients, specialists and healthcare policy makers.

## Supplementary Information

**Additional file 1.**

## Data Availability

At the end of study, (anonymized) data can be made available on request to the corresponding author. The data collected for this study will be stored up to 15 years after the end of study. This time period will take into account possible national and international legal restrictions (i.e. from the Netherlands, E.U.).

## Abbreviations

GP: General Practitioner
NoMAD: Normalisation MeAsure Development
NPT: Normalisation Process Theory
SCP: Survival Care Plan
