# Supplementary material for: Delivering colon cancer survivorship care in primary care; a qualitative study on the experiences of general practitioners
Source: BMC Prim Care. 2022 Jan 17;23:13. doi: 10.1186/s12875-021-01610-w (PMC8761520; doi:10.1186/s12875-021-01610-w)
Supplement: Supplementary file 1 — Additional file 1. [file 12875_2021_1610_MOESM1_ESM.docx]

**Supplement S1**

**Interview guide for the semi-structured interviews after 1 year of survivorship care**

Introduction

This is the interview with participant X. I would like to ask you some questions about the survivorship care you have been delivering to one of your patients with colon cancer. First we will discuss how the delivery of care was done and if you experienced any difficulties during the process. Afterwards, we will talk a bit more about the future role of the GP.

Do you consent to discussing this with me and having the conversation recorded?

Questions about the period prior to the start of the study

Can you remember how the colon cancer was diagnosed with this particular patient?

Were you involved in the diagnosis?

Were you involved during the treatment of the colon cancer?

Apart from the diagnosis of cancer, did you see this patient often?

Questions about the survivorship care

What is it like for you to deliver survivorship care to your patient?

- *Do you experience any difficulties? Which part of the process is going well and which part needs a bit more attention?*
- *How often do you see your patient during follow-up? What do you discuss during consultations? Does this change over time?*
- *Do you feel engaged with the disease process of your patient?*

How do you organize the survivorship care within the practice?

- *How much time do you have or plan for a consultation?*
- *Do you plan survivorship care separately or combine it with other care?*
- *Do you discuss psychosocial wellbeing during the consultations? Who brings possible issues forward?*
- *Has your patient had any symptoms? How do you manage these? Do you refer patients back to the specialist for symptoms?*
- *Do you feel like you have a better grasp on specific problems because you knew the patient prior to the diagnosis of colon cancer?*
- *Studies have shown that patients with cancer often consult with the GP for unrelated issues due to their fear of recurrence. Do you notice this tendency in your patient?*
- *How do you plan the consultations? Who is responsible for monitoring the care process? What is your opinion on this?*
- *Are other colleagues in the practice aware of the patients’ participation in I CARE?*

*Do you have any advice for other GPs that are just starting to deliver survivorship care?*

- *Are there any consultations outside of the follow-up schedule that are related to cancer?*

How is the communication with the specialist?

- *How was the transfer of care from specialist to GP?*
- *Is it possible to consult with the specialist?*

Do you feel the need for any additional information/support/schooling in order to deliver survivorship care?

- *Do you miss anything? How do you manage a possible shortcoming?*
- *Are you ever confronted with questions of cancer patients on which you don’t have the answers? If so, could you give an example?*

Questions about the future

How do you perceive the future role of the GP in survivorship care?

- *Would you be willing to take over survivorship care for all colon cancer patients? If so, why (not)? Are there any conditions under which this willingness applies?*
- *How is the time investment to deliver care in relation to your usual work?*
- *Do you think GPs should receive financial compensation for the delivery of survivorship care?*
- *In this study, the GP takes over survivorship care shortly after curative treatment. What is your experience with this? What do you think about the timing?*
- *Does the delivery of care influence the contact you have had with other cancer patients? Does it change your role or engagement with other cancer patients? Are you often involved with cancer patients in your practice?*

Do you have any other remarks about the care process that we have not yet discussed?

**Supplement S2**

**Interview guide for the semi-structured interviews after 5 years of survivorship care**

Introduction

For the past 5 years you have participated in the I CARE-study during which you have delivered survivorship care to one of your patients with colon cancer. This survivorship care constituted a new practice as it is usually done by the surgeon in the hospital. Through this interview, I would like to map out how the delivery of care was done and how you experienced it. The interview is anonymous and will become a part of the I CARE-study process evaluation. Do you consent to discussing this with me and having the conversation recorded?

General questions

I would like to begin with a few general questions to map out the context in which the aftercare took place.

What is your age?

How long have you been working as a general practitioner (GP)?

How long have you been working within this practice?

Is this your own practice or are you employed here?

How many staff members does the practice have?

How many patients are part of the practice?

Besides your work as a GP, do you hold any additional health-care related functions?

Questions based on NPT and CFIR constructs

Now we’ll dive into the care process itself, how this went and how it was experienced within your practice.

| **Question** | **NPT*** |
| --- | --- |
| What was it like to participate in the I CARE study? | Reflexive monitoring |
| - *What were positive and negative aspects regarding the delivery of care?* |  |
| We’re glad you agreed to participate in the I CARE study. Was this an obvious choice for you or did you have doubts? | Coherence, cognitive participation |
| - *Was it your own decision to participate or did you feel imposed by another?* |  |
| What was your motivation to participate? | Coherence, cognitive participation |
| - *Is that the main motivation? Is there any other?* |  |
| Did you have any contact with your patient before participation in this study? | Coherence, collective action |
| - *What was your role like within the care process? Did it meet your expectations?* |  |
| - *Do you feel like your role changed throughout the process?* |  |
| - *Did you discuss the care process with your patient prior to starting?* |  |
| - *How would you ideally view the patient’s role in the care process?* |  |
| How was survivorship care put into practice? | Cognitive participation, collective action |
| - *Were there any other caregivers involved with the care process? If so, how was the teamwork?* |  |
| - *Were locums and/or other GPs aware of the study?* |  |
| - *Did you discuss the study with other colleagues within the general practice?* |  |
| - *Did any other GPs have patients in the study? If so, do you know how they put this into practice?* |  |
| - *Who made the appointments and follow-up appointments?* |  |
| - *Who requested follow-up testing? Were the follow-up tests (lab and imaging) easily obtainable?* |  |
| - *How did you find the division of labour within the care process?* |  |
| - *Did the patient have a primary contact person within the general practice?* |  |
| A guideline was established for the delivery of survivorship care. | Coherence, collective action |
| - *Did you use the guideline?* |  |
| - *Which aspects of the guideline were important to you?* |  |
| - *Was the guideline sufficient to structure the care process?* |  |
| - *Did the consultations allow for discussion of psychosocial or preventive aspects of care? For example, the lifestyle and nutritional habits of the patient.* |  |
| - *Would you have liked additional schooling, or did you need any additional information?* |  |
| - *Did you feel the need to inquire about the care process? If so, where did you direct your questions?* |  |
| Did you encounter any difficulties during the delivery of survivorship care? | Collective action, reflexive monitoring |
| - *If so, which difficulties did you encounter?* |  |
| - *How did you manage abnormal follow-up test results?* |  |
| - *How was it for you to interpret the CEA values? How did you handle it? Did you ever have to repeat the test because you had any doubts?* |  |
| Did you notice any difference in the contact with your patient compared to that with other colon cancer patients? | Reflexive monitoring |
| - *Did you feel more involved with the disease process?* |  |
| Have you had any contact with the treating surgeon throughout the care process? | Collective action |
| - *If so, how did that go? Which medium did you use to communicate?* |  |
| In case survivorship care for colon cancer patients were to transfer from the surgeon to the GP completely, how many patients would your practice be able to deliver care for? | Reflexive monitoring |
| - *Would there need to be any compensation?* |  |
| Do you believe it makes sense to continue survivorship care by a GP? | Cognitive participation, reflexive monitoring |
| - *If so, why? Would you recommend to other colleagues to deliver survivorship care? Would it be possible to deliver this kind of care to all patients who have been treated for colon cancer?* |  |
| - *If not, what makes that you do not view this positively?* |  |
| What are the possible advantages and disadvantages of this form of survivorship care? | Coherence |
| Do you see any objections to survivorship care by a GP? | Cognitive participation, reflexive monitoring |
| Looking back on the study, would you have done anything differently? | Reflexive monitoring |
| - *Would you have liked to include other caregivers (practice nurse, psychologist…) in the care process?* |  |
| Did you evaluate the care process during the study with your patient or within your team or with the treating surgeon? | Reflexive monitoring |
| - *If so, how did you perform the evaluation and what did it bring to light?* |  |
| - *Did you discuss the care process at the end of the study? If so, what did it bring to light?* |  |
| I’ve asked you a lot of questions about the survivorship care. Do you have any other remarks about the care process that we have not yet discussed? |  |

* Overlap between constructs exists. Only the most important aspects are mentioned in this table.

Additional section on Oncokompas in case the patient had randomised for access to Oncokompas

As the final part of this interview, I would like to discuss the use eHealth applications in cancer survivorship care. eHealth applications are digital applications that patients may use in order to increase their own control on the care process and self-management skills.

Are you familiar with any eHealth applications in oncologic care?

Are you familiar with Oncokompas?

- *Did you discuss Oncokompas with your patient?*
- *Did you have any prior expectations about Oncokompas?*
- *Did Oncokompas ever prompted a consultation request from your patient?*
- *Do you think that Oncokompas adds to the care process? If so, why (not)?*
- *Did you ever use Oncokompas yourself?*
- *In the future, would you recommend using Oncokompas to your patient as part of the care process?*

**Supplement S3**

**COREQ (COnsolidated criteria for REporting Qualitative research) Checklist**

Developed from: Tong A, Sainsbury P, Craig J. Consolidated criteria for reporting qualitative research (COREQ): a 32-item checklist for interviews and focus groups. International Journal for Quality in Health Care. 2007. Volume 19, Number 6: pp. 349 – 357.

| **Topic** | **Item No.** | **Guide Questions/Description** | **Reported on**  **Page No.** |
| --- | --- | --- | --- |
| **Domain 1: Research team**  **and reﬂexivity** | | | |
| *Personal characteristics* | | | |
| Interviewer/facilitator | 1 | Which author/s conducted the interview or focus group? | 5 |
| Credentials | 2 | What were the researcher’s credentials? E.g. PhD, MD | 5 |
| Occupation | 3 | What was their occupation at the time of the study? | 5 |
| Gender | 4 | Was the researcher male or female? | 5 |
| Experience and training | 5 | What experience or training did the researcher have? | 5 |
| *Relationship with*  *participants* | | | |
| Relationship established | 6 | Was a relationship established prior to study commencement? | 5 |
| Participant knowledge of  the interviewer | 7 | What did the participants know about the researcher? e.g. personal  goals, reasons for doing the research |  |
|  |  |  | 5 |
|  |  |  |  |
| Interviewer characteristics | 8 | What characteristics were reported about the interviewer/facilitator? e.g. Bias, assumptions, reasons and interests in the research topic |  |
|  |  |  | N/A |
|  |  |  |  |
| **Domain 2: Study design** | | | |
| *Theoretical framework* | | | |
| Methodological orientation and Theory | 9 | What methodological orientation was stated to underpin the study? e.g. grounded theory, discourse analysis, ethnography, phenomenology, content analysis |  |
|  |  |  | 3-4 and 5 |
|  |  |  |  |
| *Participant selection* | | | |
| Sampling | 10 | How were participants selected? e.g. purposive, convenience, consecutive, snowball |  |
|  |  |  | 5 |
|  |  |  |  |
| Method of approach | 11 | How were participants approached? e.g. face-to-face, telephone, mail, email |  |
|  |  |  | 5 |
|  |  |  |  |
| Sample size | 12 | How many participants were in the study? | 5 and 6 |
| Non-participation | 13 | How many people refused to participate or dropped out? Reasons? | 5 |
| *Setting* | | | |
| Setting of data collection | 14 | Where was the data collected? e.g. home, clinic, workplace | 6 |
| Presence of non-  participants | 15 | Was anyone else present besides the participants and researchers? |  |
|  |  |  | N/A |
|  |  |  |  |
| Description of sample | 16 | What are the important characteristics of the sample? e.g. demographic data, date |  |
|  |  |  | 6-7 |
|  |  |  |  |
| *Data collection* | | | |
| Interview guide | 17 | Were questions, prompts, guides provided by the authors? Was it pilot tested? | 5, appendix S1-S2 |
|  |  |  |  |
| Repeat interviews | 18 | Were repeat inter views carried out? If yes, how many? | 7 |
| Audio/visual recording | 19 | Did the research use audio or visual recording to collect the data? | 6 |
| Field notes | 20 | Were ﬁeld notes made during and/or after the inter view or focus group? | 6 |
| Duration | 21 | What was the duration of the inter views or focus group? | 6 |
| Data saturation | 22 | Was data saturation discussed? | 6 |
| Transcripts returned | 23 | Were transcripts returned to participants for comment and/or correction? | N/A |
| **Domain 3: analysis and**  **ﬁndings** | | | |
| *Data analysis* | | | |
| Number of data coders | 24 | How many data coders coded the data? | 6 |
| Description of the coding  tree | 25 | Did authors provide a description of the coding tree? | N/A |
| Derivation of themes | 26 | Were themes identiﬁed in advance or derived from the data? | 6 |
| Software | 27 | What software, if applicable, was used to manage the data? | 6 |
| Participant checking | 28 | Did participants provide feedback on the ﬁndings? | N/A |
| *Reporting* | | | |
| Quotations presented | 29 | Were participant quotations presented to illustrate the themes/ﬁndings? Was each quotation identiﬁed? e.g. participant number | 7-14 |
| Data and ﬁndings consistent | 30 | Was there consistency between the data presented and the ﬁndings? | 7-12 (NPT) |
| Clarity of major themes | 31 | Were major themes clearly presented in the ﬁndings? | 7-12 (NPT) |
| Clarity of minor themes | 32 | Is there a description of diverse cases or discussion of minor themes? | 7-12 (NPT) |
